# Together TO-CARE: A Novel Tool for Measuring Caregiver Involvement and Parental Relational Engagement

**DOI:** 10.3390/children12081007

**Published:** 2025-07-31

**Authors:** Anna Insalaco, Natascia Bertoncelli, Luca Bedetti, Anna Cinzia Cosimo, Alessandra Boncompagni, Federica Cipolli, Alberto Berardi, Licia Lugli

**Affiliations:** 1 Department of Medical and Surgical Sciences for Mother, Children and Adults, Postgraduate School of Pediatrics, University of Modena and Reggio Emilia, 41124 Modena, Italy; 2 Neonatal Intensive Care Unit, Women’s and Children’s Health Department, University Hospital of Modena, 41100 Modena, Italy

**Keywords:** preterm infants, caregiver involvement, family integrated care, NICU

## Abstract

**Background:** Preterm infants and their families face a challenging experience during their stay in the neonatal intensive care unit (NICU). Family-centered care emphasizes the importance of welcoming parents, involving them in their baby’s daily care, and supporting the development of parenting skills. NICU staff should support parents in understanding their baby’s needs and in strengthening the parent–infant bond. Although many tools outline what parents should learn, there is a limited structured framework to monitor their involvement in the infant’s care. Tracking parental participation in daily caregiving activities could support professionals in effectively guiding families, ensuring a smoother transition to discharge. **Aims:** The aim of this study was to evaluate the adherence to and effectiveness of a structured tool for parental involvement in the NICU. This tool serves several key purposes: to track the progression and timing of parents’ autonomy in caring for their baby, to support parents in building caregiving competencies before discharge, and to standardize the approach of NICU professionals in promoting both infant care and family engagement. **Methods:** A structured template form for documenting parental involvement (“together TO-CARE template”, TTCT) was integrated into the computerized chart adopted in the NICU of Modena. Nurses were asked to complete the TTCT at each shift. The template included the following assessment items: parental presence; type of contact with the baby (touch; voice; skin-to-skin); parental involvement in care activities (diaper changing; gavage feeding; bottle feeding; breast feeding); and level of autonomy in care (observer; supported by nurse; autonomous). We evaluated TTCT uploaded data for very low birth weight (VLBW) preterm infants admitted in the Modena NICU between 1 January 2023 and 31 December 2024. Staff compliance in filling out the TTCT was assessed. The timing at which parents achieved autonomy in different care tasks was also measured. **Results:** The TTCT was completed with an average of one entry per day, during the NICU stay. Parents reached full autonomy in diaper changing at a mean of 21.1 ± 15.3 days and in bottle feeding at a mean of 48.0 ± 22.4 days after admission. The mean length of hospitalization was 53 ± 38 days. **Conclusions:** The adoption of the TTCT in the NICU is feasible and should become a central component of care for preterm infants. It promotes family-centered care by addressing the needs of both the baby and the family. Encouraging early and progressive parental involvement enhances parenting skills, builds confidence, and may help reduce post-discharge complications and readmissions. Furthermore, the use of a standardized template aims to foster consistency among NICU staff, reduce disparities in care delivery, and strengthen the support provided to families of preterm infants.

## 1. Introduction

Preterm infants and their families face a challenging experience in the neonatal intensive care unit (NICU). Infants born prematurely are at increased risk of atypical neurodevelopment, influenced by environmental factors, clinical conditions, and the stress of separation soon after birth. For parents, the journey is fraught with anxiety about their baby’s health, emotional strain from being apart, concerns about their baby’s appearance and behavior, and the overwhelming high-tech environment of the NICU [1]. Engaging parents in the NICU clinical care process may benefit the infant’s neurodevelopment [2,3]. Parent-partnered neonatal care involves parent-delivered interventions as part of daily care within the hospital. These interventions not only target the infants but also encourage active parental involvement, often offering psychological and physical benefits to the parents. Education and ongoing communication with the clinical team are typically required [4]. Infant- and Family-Centered Developmental Care (IFCDC) is a framework for newborn care that integrates neurodevelopmental and neuro-behavioral theories. IFCDC aims to provide responsive care that addresses infant behavioral cues, protects sleep, fosters parent engagement, and encourages active participation, helping parents develop essential parenting skills [5]. As part of developmental care assessment, the evidence-based, parent-friendly care pathway (i-Rainbow) was designed to meet the needs of critically ill infants in the NICU. This pathway enables caregivers to offer appropriate sensory experiences and developmental activities tailored to address the dysmaturity of preterm brains [6]. Parental presence and infant holding have been associated with improved early neurobehavioral outcomes, stronger parent–infant bonding, and healthier attachment [7,8]. The CO-PARTNER tool evaluates parent participation and collaboration in neonatal care, focusing on aspects such as daily care routines, medical care, information acquisition, advocacy, time spent with the infant, and providing comfort during hospitalization. Developed based on participation theory and parent engagement methods, this tool can motivate healthcare organizations to implement parent-delivered and parent support interventions in the NICU [9]. Although checklists and self-report instruments have been developed to guide parents in caring for their babies in the NICU, only a few tools are available to monitor and track their active involvement in daily care routines [10,11]. Even if tools such as the Parental Participation in Care: Neonatal Intensive Care Unit (PPCS:NICU) PCS:NICU have been developed and validated for the Italian context, there is still a lack of structured instruments that provide longitudinal tracking of parental autonomy in specific daily care activities throughout hospitalization, particularly for parents of VLBW and ELBW infants [12,13]. A systematic approach to tracking parent engagement could offer significant benefits. It would enable healthcare professionals to provide more effective guidance, facilitate smoother transitions home, and deepen parents’ understanding of their infant’s subtle cues and evolving needs.

In this study, we developed and adopted the Together TO-CARE template (TTCT), designed to evaluate parents’ involvement in the care of their baby during NICU hospitalization. Additionally, we assessed the compliance of NICU staff in using this tool.

## 2. Materials and Methods

### 2.1. Aim

The aim of the study was to develop and implement a tool called the Together TO-CARE template, designed to evaluate parental involvement in the care of their baby during NICU hospitalization. A secondary objective was to assess the compliance of NICU staff in using this tool.

### 2.2. Study Design

This single-center observational study was conducted from 1 January to 31 December 2024, in the Neonatal Intensive Care Unit (NICU) of Modena University Hospital.

VLBW infants admitted to the NICU at Modena University Hospital between 1 January 2023 and 31 December 2024 were considered eligible for inclusion. Exclusion criteria were outborn birth, transfer to another facility, death during hospitalization, or a length of stay of less than five days. The standard care practices in the NICU of Modena are grounded in the principles of IFCDC, with the Newborn Individualized Developmental Care and Assessment Program (NIDCAP) as a method to address the neurodevelopmental needs of both infants and parents [1,14].

Parental presence is strongly encouraged, with no restrictions on the duration or timing of their stay in the unit.

### 2.3. Creation and Use of the “Together TO-CARE Template”

The TTCT was developed and integrated into the computerized clinical chart used by NICU staff during daily care. The template was designed and structured by the multidisciplinary team dedicated to IFCDC, and it was based on existing items already in use within the NICU. The final version was implemented in clinical practice at the start of 2023 (Table 1). The TTCT tracks parental presence, contact with the infant, and kangaroo care (KC) and evaluates the autonomy of parents in various aspects of daily care (hygiene, feeding, and drug administration).

NICU staff were introduced to the tool through dedicated educational sessions. Nurses were asked to complete the TTCT during each shift. The compilation rate (defined as the number of days with at least one completed TTCT divided by the total length of hospitalization) was evaluated.

Moreover, we specifically analyzed TTCT data to assess the time required for parents to achieve autonomy in two key care tasks: diaper changing and bottle feeding.

### 2.4. Data Analysis

The distribution of continuous variables was assessed using the Shapiro–Wilk test. Variables with a normal distribution were presented as mean and standard deviation (SD), while those not normally distributed were reported as median and interquartile range (IQR).

Categorical variables were expressed as counts and percentages. The Student’s *t*-test for independent samples was used to compare continuous variables with normal distribution between two groups. The Mann–Whitney U test was used for non-normally distributed variables, as appropriate. The chi-square test was used for categorical variables. Pearson’s correlation was used to assess the relationship between continuous variables with normal distribution.

Bivariate and multivariate linear regression models were applied to explore associations between compilation rate, parental presence, and time to autonomy in neonatal care. A *p*-value < 0.05 was considered statistically significant. All statistical analyses were performed using MedCalc statistical software (version 22.007, July 2023) and Microsoft Excel.

### 2.5. Ethics

The Ethics Committee of the University Hospital of Modena granted approval for this study (ID 1839, 7 September 2021).

## 3. Results

### 3.1. Participant Characteristics

**Figure 1:**
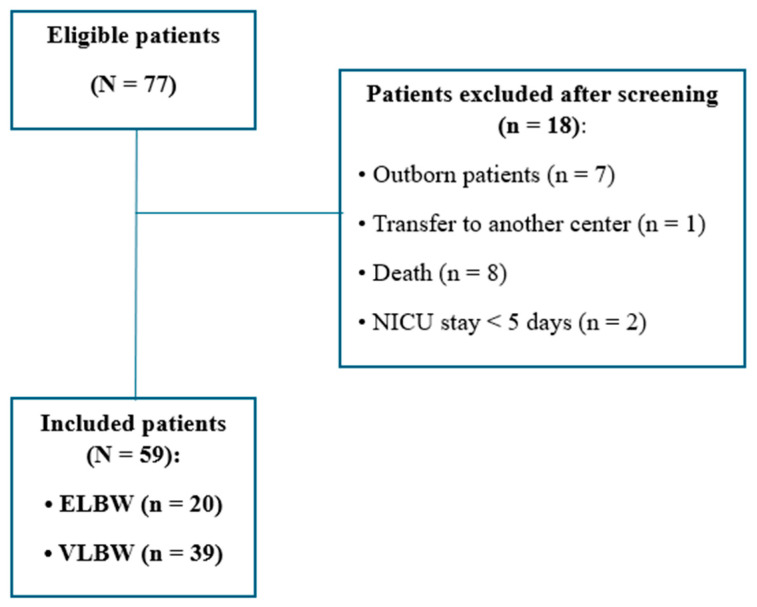
Study flowchart.

Fifty-nine VLBW infants were included in the study (Figure 1). Participant characteristics are presented in Table 2. The mean gestational age at birth was 29.5 ± 2.5 weeks, and the mean birth weight was 1138 ± 282 g. Approximately one-third (34%) were extremely low birth weight (ELBW) and 22% were classified as small for gestational age (SGA). The cohort included 33 boys (56%) and 26 girls (44%). Most infants were singletons (85%) and first-born (64%).

Most infants were Caucasian (75%). The mean maternal and paternal ages were 34.5 ± 5.8 and 37.3 ± 6.6 years, respectively. A language barrier was reported in 20% of families. Almost one-third of the mothers were unemployed at the time of delivery. The median distance from home to hospital was 17 km (range: 1.5–58).

The mean length of hospitalization was 62.0 ± 35.0 days. At discharge, the mean post-conceptional age was 38.5 ± 3.5 weeks, and the mean weight was 2468 ± 684 g.

### 3.2. Compilation Rate

The TTCT compilation rate was 67.0 ± 14%. The TTCT was mostly (64%) completed during daily shifts. Compilation rate was not significantly influenced by sociocultural factors, including language barrier and maternal occupation, nor by gestational age and birth weight (Table 3). Moreover, no statistically significant difference was found between the compilation rate during the first two weeks of life and the overall compilation rate (*p* = 0.09).

### 3.3. Caregiver Presence

The recorded average parental presence was 2.9 ± 1.0 h per day (range 1.1–5.2 h). In the bivariate analysis, maternal unemployment and language barrier were significantly associated with reduced parental presence. Families experiencing a language barrier demonstrated a significantly lower average daily presence than those without a language barrier (2.20 ± 0.8 vs. 3.09 ± 0.9 h per day; *p* < 0.01). Similarly, unemployed mothers were present significantly shorter than employed mothers (2.0 ± 0.4 vs. 3.2 ± 0.9; *p* < 0.01). However, in the multivariate linear regression, only maternal employment status remained a significant predictor of parental presence, with unemployed mothers being significantly less present during hospitalization (Table 4).

When analyzing the patterns of caregiver participation, distinguishing whether the infant was predominantly cared for by the mother alone, the father alone, or both parents together, we found that parents were predominantly present together in 69% of cases, while maternal presence was predominant in 29% of cases.

No significant differences were observed between primiparous and non-primiparous parents in terms of daily parental presence (*p* = 0.20). However, primiparous parents were more frequently present together, while non-primiparous parents were more often present separately (χ^2^ = 7.35, *p* < 0.01).

### 3.4. Caregiver Involvement and Autonomy in Daily Care

The timing of parental achievement of autonomy in neonatal care tasks is summarized in Table 5. Specifically, parents reached full autonomy in diaper changing and bottle feeding after an average of 21.1 ± 15.3 and 48.0 ± 22.4 days of life, respectively.

At discharge, almost half of the babies received breastfeeding. A significant correlation was observed between the timing of first kangaroo care and the initiation of breast approach (β coefficient = 0.42, 95% CI 0.16 to 0.63, *p* = 0.04).

**Figure 2:**
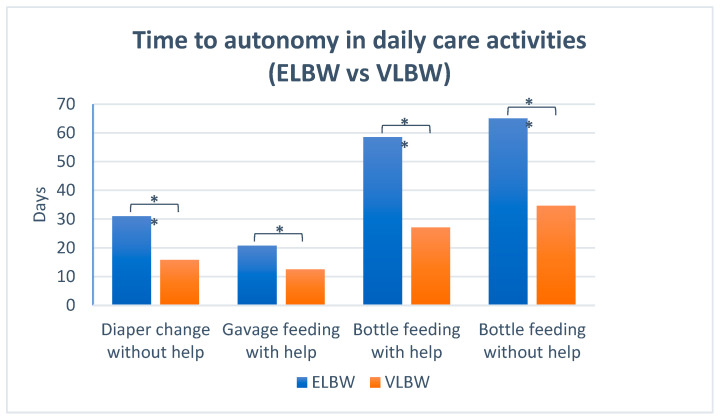
Mean time in days to reach autonomy in daily care activities in ELBW and VLBW participants. *: *p* < 0.05.

When comparing ELBW and VLBW groups, parents of ELBW infants required significantly more days to achieve full autonomy in diaper changing (31 vs. 15.8 days, *p* = 0.003) and bottle feeding (65 vs. 34.7 days, *p* < 0.001). Similar trends were observed for the first KC (7.3 vs. 4.5 days; *p* = 0.02) (Figure 2).

The delayed acquisition of autonomy in bottle feeding among ELBW infants remained evident even when considering corrected post-menstrual age (36.9 vs. 35.2 weeks; *p* = 0.002).

## 4. Discussion

This study provides insight into parental involvement in caregiving activities using the TTCT, a tool integrated into the NICU’s electronic clinical chart.

Unlike previously published instruments, which primarily focus on self-reported measures of parental self-esteem and perceived autonomy, the TTCT provides an objective and continuous assessment conducted by healthcare professionals during routine care [10,15,16,17]. While parent-reported tools offer valuable insight into the parental perspective and emotional readiness, they often lack the ability to capture day-to-day progression. On the other hand, the TTCT allows for real-time, longitudinal tracking of both parental presence and autonomy, aiming to fill a gap in current NICU practices. Previous studies have shown a decrease in parental satisfaction related to participation in care, highlighting the need to support and actively promote parental involvement [18]. To our knowledge, although some studies have addressed parental presence and participation in Italian NICUs, few have focused specifically on the longitudinal assessment of both presence and autonomy achievement in daily care tasks, particularly in the population of VLBW and ELBW infants [16,19]. A recent Italian study explored nurses’ perceptions of parental presence and involvement in the NICU using the PPCS:NICU tool [19]. While our findings align with theirs in reinforcing the need to promote early and sustained parental involvement, we used a different tool to longitudinally capture both parental presence and the progression of caregiver autonomy This allowed us to assess how parental competence evolves over time in relation to specific aspects of newborn care.

Moreover, existing data from other countries may not be directly applicable to the Italian context due to differences in social policies, economic resources, and healthcare settings [7,10,20]. However, Buccione et al. provide valuable insight into the organizational barriers that negatively affected parental presence in an Italian NICU, particularly during the COVID-19 era. The barriers reported included restrictive visiting policies, inadequate staff training in family-centered care, and limited physical space [19].

Our study highlighted the essential role of parental involvement while demonstrating the feasibility of implementing the TTCT within routine electronic documentation, supported by a consistently high completion rate. One of the strengths of the tool is its ability to collect objective data regardless of parental language barriers, thereby ensuring equitable representation of families who are often excluded from studies relying on parent-reported measures. This is particularly relevant considering that a high proportion of preterm infants are born to immigrant mothers [11,21,22]. Families with a language barrier were significantly represented (20% of the entire population of the study), and encouragingly, the compilation rate did not vary significantly by ethnicity or language. Furthermore, the completion rate during the first two weeks of hospitalization did not differ from the overall rate observed across the full length of stay. This consistency further supports the reliability of the tool in everyday NICU settings. Interestingly, in nearly 70% of cases, both parents were primarily present together, reflecting the growing trend in paternal involvement [10,23,24,25]. Our data did not show reduced presence among parents with other children, as previously reported. However, these parents were more often present separately [10,26,27,28]. Even if previous studies did not identify significant associations between maternal age, employment status, education level, or ethnicity and parental presence, our study identified maternal employment as the only significant predictor of parental presence [10,28]. Unexpectedly, maternal unemployment was associated with lower maternal presence in the NICU. This could be explained by the disadvantaged socioeconomic status of unemployed mothers, which may lead to difficulties in reaching the NICU due to transportation issues, limited autonomy, the need for accompaniment, or other factors. Conversely, employed mothers may possess a higher sociocultural status, which could facilitate their ability to visit the ward, especially as they benefit from the maternity leave provided in Italy following childbirth. Limited data are available in the literature regarding parental involvement and autonomy in daily care activities. Kim et al. reported that most parents are involved in care activities within the first week of life, while Zores et al. highlighted that many parents typically require nursing support during the first 19 days of life [10,20]. Diaper-changing autonomy is usually reached by day 26, although assistance with feeding may still be needed thereafter [20].

To our knowledge, our study is the first to document the timing of autonomy acquisition using a dedicated daily tool. In our sample, parents reached diaper-changing autonomy at a mean of 21.1 ± 15.3 days and feeding autonomy at 48.0 ± 22.4 days of life. Additionally, more than half (56%) of parents were involved in diaper changes with nursing support within the first week of life, and 83% performed KC in the same period, in line with previously published data [10]. Earlier initiation of KC was associated with earlier breast approach, suggesting that early parent–infant contact may play a role in facilitating the transition toward breastfeeding and fostering emotional closeness. Parents of VLBW infants achieved autonomy earlier than those of ELBW infants, given the generally more stable clinical condition. This finding aligns with the slower progression observed by Zores et al. among parents of extremely preterm infants [20]. As previously reported, the autonomy progression was not affected by the distance from home or other sociocultural factors [20]. Moreover, families facing language barriers achieved autonomy at similar rates compared to other families. This underscores both the strength of their engagement and the NICU staff’s efforts to actively involve them.

One limitation of our study is that it is monocentric, reflecting the practices of only one center. Moreover, recorded parental presence was quite low, especially considering that our NICU promotes IFCC. Although we cannot exclude that this data may be underestimated due to limited reporting by the staff, this study highlights the need to implement further strategies to promote prolonged parental presence and their involvement in care. Anyway, even if other European and Canadian centers report higher parental presence, up to 22 h per day, our values are comparable to those reported in other NICUs in Italy, the United States, and the Netherlands (3–4 h/day) [7,8,10,26].

## 5. Conclusions

In the context of IFCDC, this study highlights the potential value of the TTCT as a structured and inclusive tool to support and monitor parental engagement and autonomy in the NICU. By providing a real-time, nurse-driven method for documenting parental participation in care, the TTCT represents a practical screening tool to identify areas where parents may require targeted support or additional guidance. Our findings show that the tool was well accepted by healthcare professionals even if its application in daily practice could be further improved. Further studies are needed to assess the generalizability and reproducibility of the TTCT across different NICU settings and populations, in order to evaluate its broader applicability in clinical practice.

In conclusion, the TTCT represents a promising strategy to support individualized, family-centered care by enabling a structured, inclusive, and continuous monitoring of parental autonomy in the NICU setting.

## Figures and Tables

**Table 1:** “Together TO-CARE template”: final version.

Parental presence	☐ Mother ☐ Father ☐ Both parents ☐ None	If absent: specify reason for absence when known
Duration of parental stay (hours)	*Insert total hours of parental presence*	
Contact with the baby	☐ Yes ☐ No Type of contact: Touch Voice Skin-to-skin	If no: specify reason for lack of contact
Kangaroo care (KC)	☐ Yes ☐ No Breast approach: ☐ Yes ☐ No	If not performed, select a reason: ☐ Neonatal critical conditions ☐ Parental difficulties ☐ Parental absence ☐ Ongoing procedure ☐ Other
Care activities		
*Hygiene*	*Diaper Changing:* ☐ Yes ☐ No *Bathing:* ☐ Yes ☐ No	*Level of autonomy:* ☐ Observer ☐ Supported ☐ Autonomous
*Feeding*	*Gavage feeding:* ☐ Yes ☐ No *Bottle feeding:* ☐ Yes ☐ No *Breastfeeding:* ☐ Yes ☐ No	*Level of autonomy:* ☐ Observer ☐ Supported ☐ Autonomous
*Medication*	*Drug administration:* ☐ Yes ☐ No	*Level of autonomy:* ☐ Observer ☐ Supported ☐ Autonomous

**Table 2:** Clinical and socio-demographic characteristics of the study participants and their families (n = 59).

**Mean gestational age** (weeks)	29.5 ± 2.5
**Weight**	
Mean birthweight (g)	1138 ± 282
Mean birthweight (SD)	−0.39 ± 1.03
Small for gestational age	13/59 (22%)
**Gender**	
Girls	26/59 (44%)
**Birth type**	
Singleton	50/59 (85%)
**Conception**	
Spontaneous conception	47/59 (80%)
**Delivery type**	
Vaginal	9/59 (15%)
**Parity**	
Primiparous	38/59 (64%)
**Ethnicity**	
Caucasian	44/59 (75%)
Asiatic	10/59 (17%)
African	5/59 (8%)
**Language barrier**	
Yes	12/57 (21%)
**Maternal age** (years)	34.5 ± 5.8
20–29	13/58 (22%)
30–39	37/58 (64%)
Over 40	8/58 (14%)
**Paternal age** (years)	37.3 ± 6.6
21–29	6/57 (11%)
30–39	34/57 (60%)
Over 40	17/57 (29%)
**Maternal highest level of education**	
High school or less	21/59 (36%)
College/University	16/59 (27%)
**Paternal highest level of education**	
High school or less	20/59 (34%)
College/University	15/59 (25%)
**Maternal employment status**	
Employed	40/57 (70%)
**Paternal employment status**	
Employed	55/56 (98%)
**Distance home–hospital** (km)	19.3 ± 13.5
**Length of hospitalization,** days	62.0 ± 35.0
**Length of stay in NICU,** days	53.0 ± 38.0
**Discharge directly from NICU**	18/59 (30%)
**Median post-conceptional age at discharge** (weeks)	37.3 (IQR: 36.3–39.3)
**Weight at discharge**	
Mean weight at discharge (SD)	−1.27 ± 1.06
Median weight at discharge (g)	2240 (IQR: 2007–2837)
Mean weekly weight gain (g/week)	148 ± 23

**Table 3:** Unadjusted bivariate linear regression models examining the association between sociodemographic variables and TTCT compilation rate. β: unstandardized regression coefficient; CI: confidence interval.

	Unadjusted Bivariate Model		
	Coef β	95%CI	*p*
**Gestational age at birth** (weeks)	0.18	−0.83 to 1.19	0.72
**Birth weight** (g)	−0.001	−0.14 to 0.14	0.88
**Length of hospital stay** (days)	0.003	−0.11 to 0.11	0.58
**Primiparous**	−0.05	−0.61 to 0.51	0.10
**Language barrier**	0.009	−0.10 to 0.10	0.85
**Maternal age** (years)	−0.001	−0.06 to 0.06	0.80
**Maternal employment status**	0.05	−0.03 to 0.13	0.26
**Distance from hospital** (km)	0.009	−0.12 to 0.12	0.48

**Table 4:** Bivariate and multivariate linear regression models exploring the association between parental presence and sociodemographic variables. β: unstandardized regression coefficient; CI: confidence interval. R^2^ = 0.31; adjusted R^2^ = 0.27.

	Unadjusted Bivariate Model			Adjusted Multivariate Model		
	Coef β	95%CI	*p*	Coef β	95%CI	*p*
**Primiparous**	0.25	−0.37 to 0.87	0.42			
**Gestational age at birth** (weeks)	−0.02	−0.14 to 0.1	0.76			
**Birth weight** (g)	−0.001	−0.01 to 0.01	0.78			
**Language barrier**	**−0.89**	**−1.53 to −0.25**	**<0.01**	−0.18	−0.91 to 0.54	0.62
**Maternal age (years)**	0.02	−0.04 to 0.08	0.40			
**Maternal employment status**	**1.14**	**0.62–1.66**	**<0.01**	**1.12**	**0.43 to 1.81**	**0.002**
**Distance from hospital** (km)	−0.65	−1.87 to 0.57	0.30			

**Table 5:** Timing of parental involvement and autonomy in care activities. Data are expressed as mean ± standard deviation. Abbreviations: KC = kangaroo care.

Activity	First Reported Using TTCT (Days of Life)	First Reported Using TTCT in Corrected Gestational Age (Weeks)
**Diaper change with help**	8.3 ± 7.1	30.4 ±2.4
**Diaper change without help**	21.1 ± 15.3	32.4 ±2.0
**Gavage feeding with help**	15.4 ± 9.8	31.4 ± 2.4
**Bottle feeding with help**	39.5 ± 21.1	35 ± 1.3
**Bottle feeding without help**	48.0 ± 22.4	36 ± 1.5
**First KC**	5.4 ± 3.6	30.2 ±2.2
**First breast approach**	22.7 ± 16.7	32.6 ± 1.7
**Breastfeeding**	38.4 ± 18.8	34.6 ± 1.6

## Data Availability

Data are available upon request to the corresponding author.
